# Efficacy and safety of haloperidol prophylaxis for delirium prevention in older medical and surgical at-risk patients acutely admitted to hospital through the emergency department: study protocol of a multicenter, randomised, double-blind, placebo-controlled clinical trial

**DOI:** 10.1186/1471-2318-14-96

**Published:** 2014-08-28

**Authors:** Edmée JM Schrijver, Oscar J de Vries, Astrid Verburg, Karola de Graaf, Pierre M Bet, Peter M van de Ven, Ad M Kamper, Sabine HA Diepeveen, Sander Anten, Andrea Siegel, Esther Kuipéri, Anne M Lagaay, Rob J van Marum, Astrid M van Strien, Leo Boelaarts, Douwe Pons, Mark HH Kramer, Prabath WB Nanayakkara

**Affiliations:** 1 Department of Internal Medicine, VU University Medical Center, Amsterdam, The Netherlands; 2 Section of Geriatrics, Department of Internal Medicine, VU University Medical Center, Amsterdam, The Netherlands; 3 Department of Clinical Pharmacology and Pharmacy, VU University Medical Center, Amsterdam, The Netherlands; 4 Department of Epidemiology and Biostatistics, VU University Medical Center, Amsterdam, The Netherlands; 5 Section of Geriatrics, Department of Internal Medicine, Isala Hospital, Zwolle, The Netherlands; 6 Section of Acute Medicine, Department of Internal Medicine, Isala Hospital, Zwolle, The Netherlands; 7 Department of Internal Medicine, Rijnland Hospital, Leiderdorp, The Netherlands; 8 Currently: specialist training in General Practice at Leiden University Medical Center, Leiden, The Netherlands; 9 Section of Geriatrics, Department of Internal Medicine, Spaarne Hospital, Hoofddorp, The Netherlands; 10 Department of Geriatric Medicine, Jeroen Bosch Hospital, ’s Hertogenbosch, The Netherlands; 11 Department of Geriatric Medicine, Medical Centre Alkmaar, Alkmaar, The Netherlands; 12 Section of Acute Medicine, Department of Internal Medicine, VU University Medical Center, Amsterdam, The Netherlands

**Keywords:** Haloperidol, Delirium, Prophylactic treatment, Older patients

## Abstract

**Background:**

Delirium is associated with substantial morbidity and mortality rates in elderly hospitalised patients, and a growing problem due to increase in life expectancy. Implementation of standardised non-pharmacological delirium prevention strategies is challenging and adherence remains low. Pharmacological delirium prevention with haloperidol, currently the drug of choice for delirium, seems promising. However, the generalisability of randomised controlled trial results is questionable since studies have only been performed in selected postoperative hip-surgery and intensive care unit patient populations. We therefore present the design of the multicenter, randomised, double-blind, placebo-controlled clinical trial on early pharmacological intervention to prevent delirium: haloperidol prophylaxis in older emergency department patients (The HARPOON study).

**Methods/Design:**

In six Dutch hospitals, at-risk patients aged 70 years or older acutely admitted through the emergency department for general medicine and surgical specialties are randomised (n = 390) for treatment with prophylactic haloperidol 1 mg or placebo twice daily for a maximum of seven consecutive days. Primary outcome measure is the incidence of in-hospital delirium within seven days of start of the study intervention, diagnosed with the Confusion Assessment Method, and the Diagnostic and Statistical Manual of Mental Disorders, fourth edition criteria for delirium. Secondary outcome measures include delirium severity and duration assessed with the Delirium Rating Scale Revised 98; number of delirium-free days; adverse events; hospital length-of-stay; all-cause mortality; new institutionalisation; (Instrumental) Activities of Daily Living assessed with the Katz Index of ADL, and Lawton IADL scale; cognitive function assessed with the Six-item Cognitive Impairment Test, and the Dutch short form Informant Questionnaire on Cognitive Decline in the Elderly. Patients will be contacted by telephone three and six months post-discharge to collect data on cognitive- and physical function, home residency, all-cause hospital admissions, and all-cause mortality.

**Discussion:**

The HARPOON study will provide relevant information on the efficacy and safety of prophylactic haloperidol treatment for in-hospital delirium and its effects on relevant clinical outcomes in elderly at-risk medical and surgical patients.

**Trial registration:**

EudraCT Number: 201100476215; ClinicalTrials.gov Identifier: NCT01530308; Dutch Clinical Trial Registry: NTR3207

## Background

Delirium is a serious and frequently occurring complication of (acute) illness in older hospitalised patients with prevalence rates of more than 30% [1]. Delirium is a neuropsychiatric syndrome characterised by a disturbance of consciousness which has an acute onset and fluctuating course, typically developing within hours and lasting for several days to weeks or even months [1-3].

In older patients, development of in-hospital delirium is associated with a range of negative patient outcomes such as increased hospital length-of-stay, long-term cognitive- and functional decline, new institutionalisation, and increased mortality [4-6], thereby also leading to substantial healthcare costs [7]. Because of the associated healthcare and economic burden, multicomponent non-pharmacological intervention strategies targeting delirium risk factors in the hospital setting have been introduced. Although they are effective in reducing delirium incidence in elderly general medicine and surgical patients [8,9], their effectiveness seems largely to depend on protocol adherence [10] which may be low due to high staff workload. In addition, non-pharmacological multicomponent hospital-based interventions do not seem to affect post-discharge outcomes such as cognitive or functional status, and nursing home placement [11]. Low-dose haloperidol prophylaxis has been shown to lower delirium incidence in older postoperative intensive care unit (ICU) patients [12,13], and duration and severity in (mainly elective) hip-surgery patients [14]. However, the effect of haloperidol prophylaxis on in-hospital delirium development and post-discharge outcomes in an older general medicine and surgical patient population has not been studied yet.

This paper describes the design of the multicenter, randomised, double-blind, placebo-controlled clinical trial to evaluate the efficacy and safety of early prophylactic treatment with low dose haloperidol on in-hospital delirium, and post-discharge outcomes in acutely admitted older medical and surgical patients.

## Methods/Design

### Research objectives

#### Primary research objective

To study the differences in in-hospital seven-day delirium incidence after acute admission for both general medicine and surgical specialties in older at-risk patients treated early with prophylactic haloperidol or placebo.

#### Secondary outcome measures

Severity and duration of delirium; number of delirium-free days; hospital length-of-stay; adverse events; all-cause mortality in-hospital and within six months post-discharge; cognitive and physical function at baseline, three, and six months post-discharge; new institutionalisation post-discharge; number of hospital admissions within six months post-discharge.

### Study design

**Figure 1:**
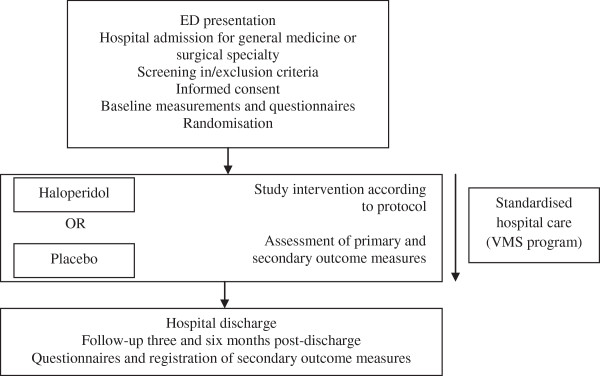
Design of the HARPOON study.

**Figure 2:**
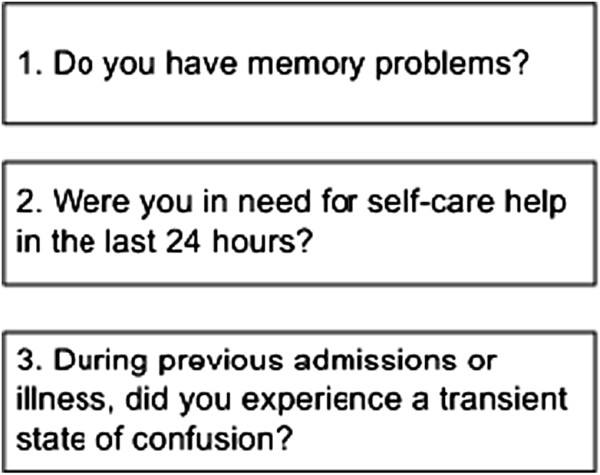
**VMS delirium risk questions.** Each positive answer values 1 point, a total score of 1 point or higher indicates the patient is at-risk for in-hospital delirium.

The HARPOON study is a multicenter, investigator initiated, stratified randomised, double-blind, placebo-controlled clinical trial with a six-month follow-up period. The study design is demonstrated in Figure 1. All at-risk patients aged 70 years or over presenting to the emergency department (ED), admitted for general medicine or surgical specialties will be screened for predefined in- and exclusion criteria as described in the section ‘Study population and recruitment’. Increased risk for developing delirium during hospital admission will be assessed according to specific delirium risk questions (Figure 2) based on the Dutch Hospital Patient Safety Program (in Dutch: VMS Veiligheidsprogramma [15]). This program was developed to systematically improve patient safety on 10 priority themes, which is now standardised hospital care in all Dutch hospitals since 2013. One of these themes is ‘Vulnerable elderly’ [15], which aims to reduce avoidable loss of function caused by complications of hospitalization and includes detection (risk assessment), and implementation of prevention and intervention measures for:

1. Malnutrition, including stimulation and measurement of nutritional intake by nutrition assistants, and dietary consultation;

2. Falls and mobility impairments, including adequate footwear, stimulation of physical activity in or out of bed, and if needed, (daily) occupational and physical therapy; and

3. Delirium.

With respect to delirium, the VMS program based standardised hospital care covers identification and modulation of precipitating delirium risk factors such as medication reviews and orientation and noise-reduction measures, creating an optimal background regimen for conduction of this trial in all participating hospitals. Patients who positively confirm one or more of the VMS delirium risk questions (VMS ≥1) are considered at-risk for developing delirium during hospital admission. Patients with VMS 0 on first evaluation are not eligible for this study. Eligible patients will be asked for informed consent. In case of inability to write, patients must provide verbal consent in the presence of an independent witness (preferably patients’ proxy) who may then sign the informed consent form. The study medication, haloperidol 1 mg or identical placebo tablets, will be given orally to participants twice daily at 12 pm and 8 pm for a maximum of seven consecutive days (14 treatment dosages) on top of standardised care based on the implemented VMS program ‘Vulnerable elderly’. During the seven-day intervention period nursing staff will screen participants for delirium symptoms. Additionally, a trained observer (physician or geriatric nurse) will daily visit participants to detect the presence of delirium, register potential adverse events based on 24-hour nursing observations, clinical judgement, and electrocardiogram (ECG) monitoring, and to check for adherence to standardised adequate environmental manipulations for delirium such as orientation measures. For patients who have not developed delirium within seven days of initiation of the study intervention, daily assessment stops. Patients who develop delirium are evaluated daily by the observer to record delirium severity and duration until symptoms diminish. Study drug administration is terminated early if a participant develops delirium, is transferred to a non-participating care unit such as the ICU, refuses further participation in the trial, the QTc interval is more than 500 milliseconds (ms), is discharged from the hospital, or dies within seven-day of initiation of the study intervention. In case of established delirium or if the assigned study intervention is thought to have a direct influence on the patient’s treatment, the treating physician will request for unblinding and may further decide on treatment. For unblinding of treatment assignment, the treating physician is able to contact the VUmc consultant pharmacist through a central telephone number 24 hours a day, seven days a week.

This study is conducted according to the Declaration of Helsinki. This trial is approved by the accredited Medical Ethical Committee of VU University Medical Center (VUmc) Amsterdam, the Netherlands (reference number: 2012.177). The executive boards of Isala Hospital Zwolle, Spaarne Hospital Hoofddorp, Rijnland Hospital Leiderdorp, Jeroen Bosch Hospital’s Hertogenbosch, and Medical Centre Alkmaar (MCA), all located in the Netherlands, have provided local feasibility approval.

### Location and setting

This study is primarily conducted at VUmc, a 733-bed teaching hospital located in Amsterdam, the Netherlands. Secondary study locations include the Isala Hospital Zwolle (976 beds), Spaarne Hospital Hoofddorp (455 beds), Rijnland Hospital Leiderdorp (420 beds), Jeroen Bosch Hospital ’s Hertogenbosch (730 beds), and MCA Alkmaar (485 beds), the Netherlands.

### Study population and recruitment

#### Inclusion criteria

Patients aged 70 years and over are eligible when acutely admitted to the hospital through the ED for general medicine or surgical specialties, are able to speak and understand Dutch or English language, are at-risk for delirium on admission (VMS ≥1), are available for inclusion within 24 hours after admission, and are able to provide informed consent. Patients are considered competent to provide informed consent if – after having spoken with the patient – the observer and/or the treating physician agree on the ability of the patient to understand and/or recall relevant study information, including the purpose of the study, its experimental nature, potential benefits and risks, the right to withdraw, and confidentiality of collected data, and to make a voluntary decision to participate. If their cognitive competence is in question, the patient will be regarded as ineligible.

#### Exclusion criteria

Exclusion criteria for participation are delirium on admission according to the DSM-IV criteria; VMS 0; patients not able to take study medication according to protocol (including nil per os); specific heart conditions on admission including QTc interval of more than 500 ms, recent myocardial infarction, decompensated heart failure, second- or third-degree AV block, (history of) ventricular arrhythmias or Torsade de Pointes (TdP), uncorrected hypokalemia with serum potassium level 3.0 milliequivalents per liter (mEq/L) or less, clinical significant bradycardia; concomitant use of pharmacodynamically interacting medication; use of antipsychotic or dopaminergic drugs; use of QT prolonging drugs in the presence of prolonged QTc interval (more than 450 ms and 460 ms, for men and women respectively) on baseline ECG; Parkinson’s Disease; Vascular or Lewy Body Dementia; Hypokinetic Movement Disorder; (history of) Neuroleptic Malignant Syndrome; Central Anticholinergic Syndrome; substance abuse and dependence according to the DSM-IV criteria; (history of) epilepsy; concomitant participation in another clinical trial with the exception of observational studies; not competent to provide informed consent.

### Randomisation

Two well-known variables associated with increased risk of delirium are age 80 years or over and surgical treatment/anaesthesia. To balance population representatives in both treatment groups, four strata are constructed with separate randomisation schedules: (1) age 70 to 80 years, planned surgery on admission; (2) age 70 to 80 years, no planned surgery on admission; (3) age 80 years or over, planned surgery on admission; (4) age 80 years or over, no planned surgery on admission. Each stratum consists of 20 pre-randomised individual study numbers, 10 assigned to haloperidol and 10 to placebo treatment, prepared by the Central Pharmacy for The Hague Hospitals (Apotheek Haagse Ziekenhuizen/AHZ located in The Hague, the Netherlands). Participants, observers, physicians, nurses, other care givers, at the investigative sites are all blinded to the assigned intervention. The VUmc clinical pharmacy holds the randomisation list.

### Study interventions

AHZ is responsible for preparing, labelling, packaging and distributing the study medication according to Good Manufacturing Procedure (GMP) guidelines. Both the haloperidol (as a verum) and placebo tablets are identical in appearance, have fixed concentrations of 1 mg per tablet, and are packaged in identically labelled medicine carton boxes. Study medication prescription is standardised in every study centre’s automated prescription system. On prescription by the local investigator(s), a carton box containing study medication (either haloperidol or placebo tablets) can be collected. To ensure study medication access 24 hours a day, seven days a week, medicine boxes are securely stored in the ED or on the wards rather than in the local clinical pharmacy.

### Data collection

Data will be mainly collected by two observers, a physician (EJMS) and geriatric nurse (AV) trained according to the Good Clinical Practice (GCP) principles. Participants’ demographic data, medical history, (prescription) medication history on admission (including the use of sleep medication and antidepressants), presence of sleep-wake cycle disturbances, visual and/or hearing impairment, substance use, and vital parameters will be documented. Medical history and (prescription) medications will be coded according to the World Health Organization’s International Statistical Classification of Diseases and Related Health Problems 10^th^ revision (ICD-10) [16], and the Anatomical Therapeutic Chemical (ATC) Classification System [17]. Severity and number of co-morbidities are listed according to the Charlson comorbidity index using the ICD-10 coding algorithm [18,19]. Physical function is assessed with the Katz Index of Independence in Activities of Daily Living (ADL) [20,21] and the Lawton Instrumental Activities of Daily Living (IADL) scale [22]. The Six Item Cognitive Impairment Test (6-CIT) is used to evaluate participants’ cognitive function [23-25]. Primary caregivers are asked to complete the IQCODE-N in order to reflect on the participant’s cognitive function prior to hospitalisation [26]. The Confusion Assessment Method (CAM) will be used to diagnose delirium according to the Diagnostic and Statistical Manual of Mental Disorders, Fourth edition (DSM-IV) delirium criteria during the study intervention period, and to ensure delirium is not present on study inclusion [27]. Nurses register the Delirium Observation Screening (DOS) scale three times a day (once every day, evening and night shift) during the intervention period to evaluate delirium symptoms [28,29]. Once delirium is established, one of the observers will perform the DRS-R-98 once-daily to assess delirium severity and duration [30]. An ECG is performed at baseline, after two and six treatment dosages, and at the end of the intervention period to register QTc intervals. ECG is repeated daily until a steady state is reached in case of prolonged QTc interval (more than 450 ms and 460 ms, for men and women respectively), or if the patient is taking other QT prolonging medications. On study inclusion, and at the time of ECG after study drug administration dose six, a 4 ml blood sample is collected, and plasma is stored at -80°C at the VUmc Clinical Pharmaceutical and Toxicological Laboratory to determine haloperidol levels. On discharge, the number of days spent in the hospital is recorded.

### Follow-up assessments

Three and six months after discharge, patients are contacted by telephone by one of the observers to perform the Katz ADL, the Lawton IADL scale, and the 6-CIT, and to obtain data on home residency, hospital admissions, (recurrent) delirium, and all-cause mortality.

### Study monitoring

This study will be monitored by two members – the manager and an independent clinical research assistant – of the Clinical Research Unit, Department of Internal Medicine, VUmc Amsterdam, the Netherlands. During the monitoring visits at respectively the beginning, half-way and end of the study, the consistency of data collection, documentation and the presence and completeness of the Investigator Site File are checked for compliance with GCP guidelines. Findings are systematically recorded and signed by the monitors.

### Data Safety Monitoring Board

Because this study is a pharmacological intervention study recruiting (vulnerable) older patients, we appointed a Data Safety Monitoring Board (DSMB). The primary mandate of the DSMB is to monitor patient safety during the trial. The DSMB for this study consists of four independent members: two emeritus professors of internal medicine, a professor of clinical epidemiology, and a consultant physician in geriatric medicine. The DSMB reviews unblinded results and convenes prior to the beginning of the study, and thereafter, every six months from the first randomisation date. On request, the DSMB may obtain unblinded study results from the hospital pharmacy. When a study participant dies, the DSMB evaluates the event and mortality rates in both study arms. After 125 patients have completed the entire study period including follow-up in each study arm, the DSMB will perform a safety interim analysis with respect to all-cause mortality.

### Sample size calculation

The primary study objective is to evaluate the effect of prophylactic haloperidol compared to placebo for the incidence of in-hospital delirium within seven days of start of the study intervention. Based on previous studies, we expect the incidence of delirium in our target patient population currently to be 20%. Aiming for an absolute reduction of 10% in the haloperidol intervention group, with the significance level set at 5%, inclusion of n = 195 patients per study arm (390 patients in total) will give us a power of 80% to detect differences with a two-sided alpha of 0.05.

### Statistics

For the data analysis IBM SPSS Statistics 20 will be used. Clinical trial data will be primarily registered in paper case report forms and later transcribed into the electronic data capture system of OpenClinica, open source clinical trail software. The primary analysis is aimed at evaluation of the primary outcome of this study: the incidence of in-hospital delirium according to the DSM-IV criteria in older at-risk patients aged 70 years or over who are acutely admitted through the ED for a general medicine or surgical specialty within seven days of initiation of the study intervention. Data from all randomised patients will be analysed according to the Intention-to-treat principal. The primary outcome delirium incidence is considered a binary outcome, present or absent. Patients who for example leave the hospital (or die in-hospital) within seven days of start of the study intervention without experiencing delirium will be regarded negative on the primary outcome. Patients who do not complete the intervention will be monitored during their in-hospital stay for occurrence of delirium. The number of patients diagnosed with delirium at least once in the seven days following initiation of the study intervention will be calculated for both the haloperidol and placebo group, and subsequently this number is divided by the total amount of patients assigned to the matching group, reflecting in-hospital delirium incidence ratio within seven days of initiation of the study intervention. Pearson’s chi-squared test will be used to compare this number between the two groups. Continuous normally distributed data including patient demographic data will be tested with Student’s *t* test. The Mann–Whitney *U* test is used for comparison of non-normal distributed data such as secondary outcome measures concerning efficacy of prophylactic haloperidol treatment. Log rank test will be used to establish the time to (first) delirium diagnosis (number of delirium-free days), which will be compared between groups. Adverse events in both groups will be globally described. Kaplan-Meier estimates will be used to display survival probability within 6 months after hospital discharge for patients with and without diagnosed in-hospital delirium.

## Discussion

This stratified randomised, placebo-controlled, double-blind clinical trial studies the efficacy and safety of haloperidol prophylaxis for prevention of delirium in older at-risk patients aged 70 years or over who are acutely admitted to the hospital through the ED for general medicine or surgical specialties, because generalisability of existing study results from well-designed RCTs in this field are limited to postoperative (mainly elective) hip-surgery [14] and ICU patients [13].

In general, in-hospital delirium occurs within three or four days after admission or surgery [12,31], but the mean time to onset may vary up to approximately six days in ICU patients [13]. Because delirium severity and duration amongst other things were chosen as secondary outcome parameters, a study intervention-period was selected that is long enough to also identify delirium developing under prophylactic haloperidol treatment. Treatment response after initiation of haloperidol treatment is to be expected within 24 to 48 hours after initiation of therapy [32,33]. A small RCT in 47 hip surgery patients aged 70 and older demonstrated that a haloperidol treatment regimen of 1 mg three times daily was more effective in reducing severity of established delirium than 1.5 mg twice-daily or 3 mg once-daily (mean highest DRS-R-98 score ± standard deviation of 15.8 ± 5.2, versus 21.3 ± 4.7, and 19.2 ± 6.1 respectively), while duration of delirium tended to be shorter in the twice-daily dosing group (2.3 ± 1.8 days, versus 3.9 ± 2.6 and 4.1 ± 1.9 days in the three times daily and once-daily group respectively). No side effects were noted in the total study population (total haloperidol dose 3 mg/day) [34]. In a large placebo-controlled RCT in 430 hip surgery patients aged 70 years and older, a three times daily prophylactic regimen with haloperidol (total dose 1.5 mg/day, maximum intervention period six days) did not significantly reduce postoperative delirium incidence in hip surgery patients, though it did significantly reduce delirium severity (mean highest DRS-R-98 score 14.4 ± 3.4 versus 18.4 ± 4.3) and duration (5.4 versus 11.8 days, 95% CI = 2.0-5.8; p < 0.001) in the haloperidol compared to the placebo group respectively [14]. Aforementioned study results suggest that although a three times daily dosing strategy with haloperidol seems superior in reducing symptom severity in the treatment of established delirium, it is not efficacious in reducing the incidence of postoperative delirium when administered prophylactic. Since the primary objective of this study is to investigate the effect of prophylactic haloperidol administration on delirium incidence in a broader elderly patient population, a different treatment regimen of 1 mg haloperidol or placebo twice-daily for a maximum intervention period of seven consecutive days was chosen. In case of established delirium, symptoms tend to fluctuate during the course of the day with potential evening or night-time worsening. Given that haloperidol peak plasma concentrations occur within two to six hours after oral administration, administration of the prophylactic study intervention in the early afternoon and mid evening was favoured. Capsule count of returned study medication in addition to evaluation of nurse medication charts confirmed by plasma drug levels is used as a reliable method of assessing study medication adherence in this clinical trial [35].

In addition to extrapyramidal side effects, more rare side effects as ventricular arrhythmia’s and TdP are a great concern for physicians initiating haloperidol treatment in a patient. To date, there are no consensus practice guidelines on QT(c) monitoring for the prevention of TdP in the hospital setting, and recommendations are primarily based on clinical experience [36-38]. During this study routine ECG measurements will be performed and plasma haloperidol levels will be determined to evaluate individual QTc changes related to these haloperidol levels in order to expand the current knowledge base.

Eligibility criteria include that a subject has the cognitive capacity to understand the scope of the study and is able to provide informed consent independently. Based on the sample size calculation, a large numbers of subjects must be screened. Therefore, a brief and valid screening instrument was selected to assess cognitive function (6-CIT) [24,25] on admission in a face-to-face interview. The Dutch version of the 6-CIT has high diagnostic accuracy compared with the cognitive impairment criterion standard Mini-Mental State Examination [23]. Although the 6-CIT has not been validated for telephone administration, we considered it to be accurate for use over the telephone given that it includes diagnostic properties (e.g. temporal orientation, and object recall) similar to a six-item screener suitable for telephone administration [39]. The Katz ADL has been shown a reliable way to collect data on ADL by telephone [40]. Telephone follow-up was chosen because it is safe and more efficient than re-inviting subjects for a face-to-face interview.

In conclusion, the HARPOON study is a well-designed double-blind, placebo-controlled RCT that we believe will provide relevant information on the efficacy and safety of early administration of low-dose haloperidol for preventing delirium in at-risk elderly patients in the hospital setting, and its effects on post discharge outcomes. The predicted study completion is May 1^st^ 2015.

## Abbreviations

6-CIT: Six-item Cognitive Impairment Test; ADL: The Katz Index of Independence of Activities of Daily Living; AHZ: Central Pharmacy for The Hague Hospitals (in Dutch: Apotheek Haagse Ziekenhuizen/AHZ, The Hague, the Netherlands); ATC: Anatomical Therapeutic Chemical (Classification System); CAM: Confusion Assessment Method; DOS: Delirium Observation Screening (scale); DRS-R-98: Delirium Rating Scale Revised 98; DSMB: Data Safety Monitoring Board; DSM-IV: Diagnostic and Statistical Manual of Mental Disorders, Fourth edition; ECG: Electrocardiogram; ED: Emergency department; EudraCT: European drug regulatory affairs clinical trials; GCP: Good Clinical Practice; GMP: Good Manufacturing Procedure; ICD-10: International Statistical Classification of Diseases and Related Health Problems 10^th^ revision; ICU: Intensive care unit; IQCODE-N: Informant Questionnaire on Cognitive Decline in the Elderly; IADL: The Lawton Instrumental Activities of Daily Living scale; MCA: Medical Centre Alkmaar; mEq/L: Milliequivalents per liter; mg: Milligrams; ml: Millilitres; ms: Milliseconds; QTc: QT interval corrected for heart rate; TdP: Torsade de Pointes; VMS: Dutch Hospital Patient Safety Program (in Dutch: Veiligheidsprogramma); VUmc: VU University Medical Center.

## Competing interests

The authors declare that they have no competing interests.

## Authors’ contributions

EJMS, OJV, PMB, and PWN conceived of the study and drafted the study protocol. EJMS, OJV, AV, KG, PMB, AMK, SHAD, SA, AS, EK, AML, RJM, AMS, LB, DP, MHHK, and PWBN participate in the design and coordination of the study. EJMS, AV, and KG support the data collection. PWBN has overall responsibility for the trial. EJMS, OJV, PMV, and PWBN performed the statistical analysis for the study protocol and the manuscript, and will primarily be responsible for interpretation of the study results and primary data analysis. EJMS drafted and revised the manuscript. OJV, PMV, PWBN critically revised the manuscript for important intellectual content. All authors have read and approved the final manuscript.

## Pre-publication history

The pre-publication history for this paper can be accessed here:

http://www.biomedcentral.com/1471-2318/14/96/prepub
